# Pre-Pregnancy Obesity Phenotypes and the Risk of Oligohydramnios: A Large Population-Based Cohort

**DOI:** 10.3390/jcm15176652

**Published:** 2026-08-28

**Authors:** Kyung Eun Lee, Ye Bin Park, In Yang Park, Jae Eun Shin, Kyung Do Han

**Affiliations:** 1 Department of Obstetrics and Gynecology, Bucheon St. Mary’s Hospital, College of Medicine, The Catholic University of Korea, Bucheon 14647, Republic of Korea; gangiii@catholic.ac.kr; 2 Department of Statistics and Actuarial Science, Soongsil University, Seoul 06978, Republic of Korea; par6799@naver.com; 3 Department of Obstetrics and Gynecology, Seoul St. Mary’s Hospital, College of Medicine, The Catholic University of Korea, Seoul 06591, Republic of Korea; ooooobbbbb@catholic.ac.kr

**Keywords:** amniotic fluid, body mass index, obesity, obesity, abdominal, oligohydramnios, pregnancy complications

## Abstract

**Background/Objective:** Maternal obesity is an established risk factor for adverse obstetric outcomes. However, its association with oligohydramnios remains unclear and has been examined using body mass index (BMI) alone. We assessed pre-pregnancy general and abdominal obesity in relation to oligohydramnios. **Methods:** We analyzed 762,104 women who delivered between 2010 and 2018 and had undergone a national health examination within two years before conception. Women were classified into four phenotypes by BMI (general obesity: ≥25 kg/m^2^) and waist circumference (abdominal obesity: ≥85 cm). Odds ratios were estimated by logistic regression adjusted for pre-pregnancy characteristics, and interaction was tested on the additive and multiplicative scales. **Results:** Oligohydramnios occurred in 13,860 women (1.82%). Relative to women with neither phenotype, adjusted odds were increased for abdominal obesity alone (1.163; 95% CI 1.013–1.336), general obesity alone (1.125; 95% CI 1.051–1.204), and both (1.336; 95% CI 1.241–1.438). Absolute risk was 2.19% among women with both phenotypes and 1.79% among those with neither, with no interaction on either scale (*p* = 0.811). **Conclusions:** General and abdominal obesity were each associated with oligohydramnios. Because nearly one in four women with abdominal obesity had a BMI below the obesity threshold, assessment by BMI alone leaves part of this risk unrecognized.

## 1. Introduction

Obesity has become a major public health concern worldwide, with rising prevalence among women of reproductive age. In South Korea, increases in both general and abdominal obesity have been particularly notable [1,2], and the increase has been steepest in women of reproductive age. Between 2012 and 2021 the prevalence of obesity among Korean women in their twenties nearly doubled, from 9.7% to 18.2%, and abdominal obesity rose more than 1.6-fold in women in their twenties and thirties [2]. Similar trends are observed in many high-income countries, where approximately one in four women begins pregnancy with obesity [3,4]. As these patterns intensify, understanding the implications of maternal obesity for perinatal outcomes has become increasingly important.

Oligohydramnios, a pathological reduction in amniotic fluid volume, complicates a small but clinically important proportion of pregnancies. Reduced amniotic fluid volume has been associated with umbilical cord compression, meconium-stained fluid, non-reassuring fetal heart rate patterns, operative delivery for fetal distress, and admission to neonatal intensive care [5,6]. The prognostic significance of reduced amniotic fluid depends on the gestational age at which it arises and on whether other complications are present. Because oligohydramnios is most often first detected on third-trimester ultrasonography, the window for preventive action is narrow, and risk factors identifiable before conception are of particular clinical interest.

Pre-pregnancy obesity is associated with a range of adverse obstetric outcomes, including gestational diabetes mellitus (GDM), hypertensive disorders of pregnancy (HDP), and an increased rate of cesarean delivery [7,8,9]. Whether maternal adiposity also influences amniotic fluid volume is less clear, and the available evidence is limited and inconsistent. One large cohort reported an increased risk of abnormal amniotic fluid volume among women with obesity in early pregnancy [10], whereas other investigations found no association between elevated BMI and oligohydramnios [11,12,13]. These studies characterized adiposity by BMI alone, which conveys total body mass but not the distribution of body fat. Central adiposity is metabolically distinct from subcutaneous fat [14], and in Korean adults, WC is associated with insulin resistance independently of BMI [15]. In obstetric research, central adiposity assessed by ultrasonographic fat thickness has been related to birth weight and to cesarean delivery [16,17], yet amniotic fluid volume has not been examined as an outcome. To our knowledge, no previous study has classified women simultaneously by general and abdominal obesity and evaluated the resulting phenotypes in relation to oligohydramnios.

This study examined the association between pre-pregnancy obesity phenotypes and the risk of oligohydramnios in a nationwide Korean cohort, using BMI and waist circumference (WC) measured at standardized national health examinations before conception. Women were classified according to the presence of general obesity, abdominal obesity, both, or neither. We hypothesized that women meeting criteria for both phenotypes would show the highest risk, and we assessed whether this joint classification identifies a subgroup at higher absolute risk of oligohydramnios.

## 2. Materials and Methods

### 2.1. Data Resource

This study utilized data from the National Health Insurance Service (NHIS) of South Korea, which provides mandatory universal health coverage for approximately 97% of the population [18]. The database includes detailed information on healthcare utilization, demographic characteristics, diagnostic and procedural codes, and results from the National Health Screening Examination (NHSE), a standardized biennial program that collects anthropometric, biochemical, and lifestyle data. Obstetric outcomes were identified from delivery-related claims data. All datasets were anonymized before analysis, and the study protocol was approved by the Institutional Review Board (IRB No. HC25ZISI0045).

### 2.2. Study Population

A total of 2,403,853 deliveries between January 2010 and December 2018 were initially identified. The onset of pregnancy was dated to 280 days before delivery, corresponding to conventional gestational dating from the last menstrual period (LMP). Women were eligible if they had undergone NHSE within the two years preceding this estimated date of the LMP. Because the LMP precedes conception by approximately two weeks, every examination included in the analysis antedates conception by at least that further margin. As the NHSE is offered biennially, this window captures the examination most proximate to conception for the majority of women.

For women with more than one delivery during the study period, the most recent delivery for which an eligible examination was available was selected as the index pregnancy, so that each woman contributes a single observation. On this basis 819,746 women were identified.

**Figure 1:**
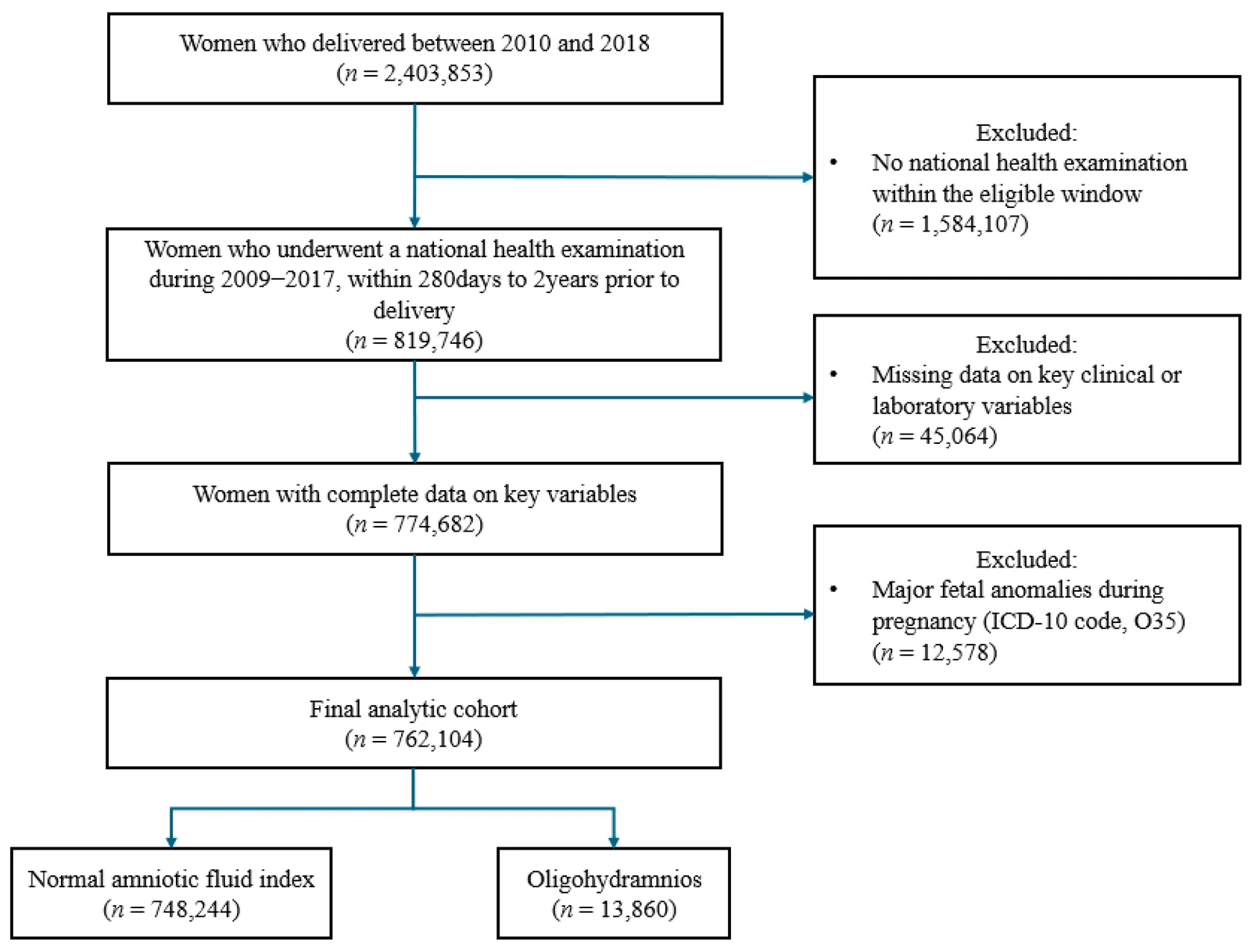
Flowchart of the study population selection.

We excluded women with incomplete demographic, anthropometric, or biochemical information (*n* = 45,064) and pregnancies complicated by fetal anomalies, identified by International Classification of Diseases, 10th Revision (ICD-10) code O35. This exclusion also removes congenital renal anomalies from the analytic population. After applying these criteria, 762,104 women were included in the final analysis (Figure 1).

### 2.3. Covariates

Maternal characteristics included maternal age (categorized as <25, 25–29, 30–34, 35–39, and ≥40 years), smoking status (never, former, current), and alcohol consumption (non-drinker, mild, heavy). Smoking status and alcohol consumption were based on self-reported NHSE questionnaires. Reproductive and gynecological histories—nulliparity, multifetal pregnancy, history of abortion, uterine myoma, adenomyosis, endometriosis, and polycystic ovarian syndrome (PCOS)—were identified through ICD-10 codes.

Pre-pregnancy metabolic comorbidities were defined by combining health examination measurements with claims records. Diabetes mellitus was defined as a fasting glucose of 126 mg/dL or higher, or a diagnosis of E11–E14 accompanied by antidiabetic medication. Hypertension was defined as a systolic blood pressure of 140 mmHg or higher, a diastolic blood pressure of 90 mmHg or higher, or a diagnosis of I10–I13 or I15 accompanied by antihypertensive medication. Dyslipidemia was defined as a total cholesterol of 240 mg/dL or higher, or a diagnosis of E78 accompanied by lipid-lowering medication. Chronic kidney disease was defined as an estimated glomerular filtration rate (eGFR) below 60 mL/min/1.73 m^2^, derived from serum creatinine measured at the examination, or receipt of dialysis before the examination. Systemic lupus erythematosus (SLE)/antiphospholipid syndrome (APS) were identified from claim records.

GDM was defined as at least two claims carrying ICD-10 code O24.4 or O24.9, or a prescription for insulin during the index pregnancy. HDP were identified by ICD-10 codes O13–O16. Blood pressure, fasting glucose, and the lipid profile (total cholesterol, high-density lipoprotein cholesterol (HDL-C), low-density lipoprotein cholesterol (LDL-C), and triglycerides) were also recorded as continuous measurements at the examination. Triglycerides were log-transformed for analysis because of their skewed distribution and are presented as geometric means. Nulliparity was defined as the absence of any previous delivery prior to the index pregnancy. Household income and regular physical activity were recorded at the examination and are presented as baseline characteristics.

### 2.4. Assessment of Pre-Pregnancy Obesity

Pre-pregnancy BMI was calculated as weight in kilograms divided by height in meters squared (kg/m^2^). BMI categories were defined as underweight (<18.5 kg/m^2^), normal (18.5–22.9), overweight (23.0–24.9), class I obese (25.0–29.9), and class II or higher obese (≥30.0). In accordance with the Korean Society for the Study of Obesity (KSSO) and World Health Organization (WHO) Asia-Pacific guidelines, BMI ≥ 25.0 kg/m^2^ was considered general obesity.

WC was categorized into six groups: <75, 75–79.9, 80–84.9, 85–89.9, 90–94.9, and ≥95 cm. Abdominal obesity was defined as WC ≥ 85 cm based on KSSO criteria, and in regression analyses, the 80–84.9 cm category served as the reference group.

### 2.5. Outcome: Oligohydramnios

The outcome was oligohydramnios, which in clinical practice is diagnosed by an amniotic fluid index (AFI) below 5 cm or a maximum vertical pocket of 2 cm or less [19,20]. Cases were ascertained from claims records as at least one claim carrying ICD-10 code O41.0 within the 280 days preceding delivery, a window corresponding to the assumed duration of gestation from the LMP. Ultrasonographic measurements are not held in the NHIS database, and the underlying sonographic parameters could not be verified.

### 2.6. Statistical Analysis

Baseline characteristics were summarized using descriptive statistics. Continuous variables were presented as means (standard deviations) and compared using ANOVA. Categorical variables were presented as counts and percentages and compared using the chi-square test.

Multivariable logistic regression was used to estimate odds ratios (ORs) and 95% confidence intervals (CIs) for oligohydramnios according to obesity indices. Model 1 was unadjusted. Model 2, the primary adjusted model, included variables antedating pregnancy: maternal age, smoking status, alcohol consumption, nulliparity, multifetal pregnancy, diabetes mellitus, hypertension, dyslipidemia, chronic kidney disease, SLE/APS, and gynecologic comorbidities (uterine myoma, adenomyosis, endometriosis, PCOS, and history of abortion). Model 3 comprised Model 2 with additional adjustment for GDM and HDP. As these conditions may lie on the causal pathway between pre-pregnancy adiposity and oligohydramnios, this model is presented as a secondary analysis. Model 4 comprised Model 2 with additional adjustment for fasting glucose and the lipid profile and is presented as an exploratory analysis.

Absolute risks and risk differences were calculated for each obesity phenotype alongside the corresponding odds ratios. Interaction between general and abdominal obesity was assessed on two scales. Multiplicative interaction was tested by a product term, with the two phenotypes entered as binary categories and, in a parallel specification, as continuous measures of BMI and WC. Additive interaction was assessed by the relative excess risk due to interaction, the attributable proportion, and the synergy index, each with 95% confidence intervals [21]. The dose–response relationship between BMI, WC, and oligohydramnios was examined using spline analysis.

Three sensitivity analyses were performed. The first stratified the cohort by the interval between the health examination and the estimated LMP, comparing women examined within one year with those examined one to two years beforehand. The second was restricted to singleton pregnancies. The third was restricted to isolated oligohydramnios, excluding premature rupture of membranes (O42), fetal growth restriction (O36.5), and post-term pregnancy (O48).

A two-sided *p*-value < 0.05 was considered significant. All statistical analyses were conducted using SAS software, version 9.4 (SAS Institute Inc., Cary, NC, USA). Spline curves were plotted using R, version 4.3.3 (R Foundation for Statistical Computing, Vienna, Austria).

## 3. Results

Among the 762,104 women included in the analysis, 13,860 (1.82%) had a diagnosis of oligohydramnios. Women with oligohydramnios were more frequently nulliparous (76.1% versus 64.6%) and less frequently carried a multifetal pregnancy. Current smoking, alcohol consumption, and PCOS were more common, and mean pre-pregnancy BMI and WC were higher, as was the prevalence of both general and abdominal obesity. Among pregnancy complications, HDP showed the largest difference (6.6% versus 2.8%), while GDM and SLE/APS were also more frequent (Table 1). The interval between the health examination and the estimated date of conception did not differ between the two groups.

Table 2 presents the associations between pre-pregnancy obesity indices and oligohydramnios. Relative to a BMI of 18.5–22.9 kg/m^2^, the adjusted odds of oligohydramnios rose progressively across categories, reaching 1.495 (95% CI 1.343–1.665) at 30.0 kg/m^2^ or above. Women with a BMI below 18.5 kg/m^2^ were not at increased risk. General obesity was associated with an adjusted odds ratio of 1.208 (1.147–1.272).

**Figure 2:**
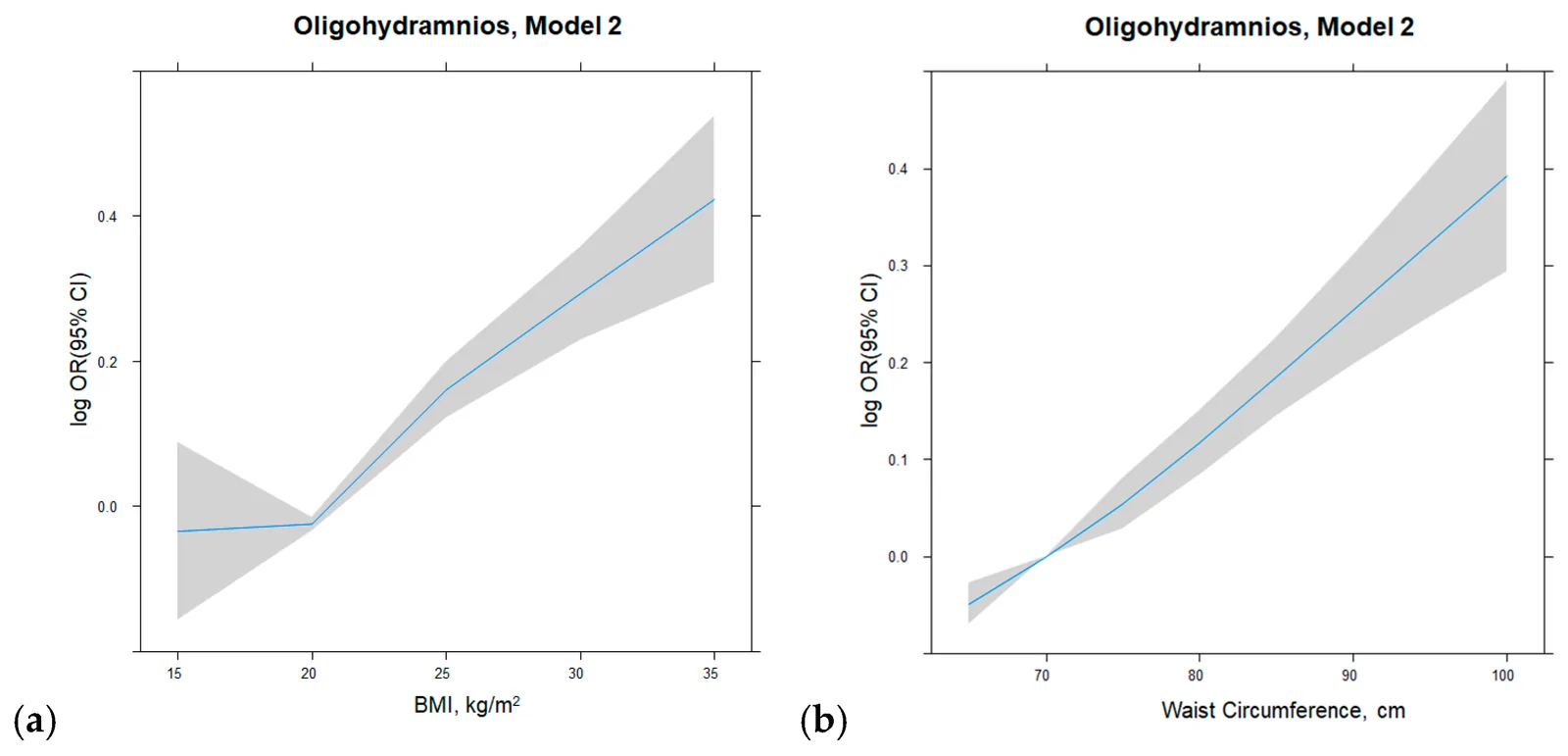
Dose–response relationship between pre-pregnancy adiposity and the risk of oligohydramnios. Log odds ratios (solid lines) with 95% confidence intervals (shaded area) for oligohydramnios across (**a**) pre-pregnancy BMI and (**b**) pre-pregnancy WC, modelled as continuous variables using spline analysis. Estimates are from Model 2. A log odds ratio of 0 corresponds to an odds ratio of 1. Abbreviations: BMI, body mass index; WC, waist circumference.

A similar gradient was observed for WC, with the highest adjusted odds ratios in the two uppermost categories relative to 80.0–84.9 cm. The estimate for 85.0–89.9 cm was close to unity, and the number of events in the uppermost categories was small. Abdominal obesity was associated with an adjusted odds ratio of 1.281 (1.199–1.369). Spline analysis showed a monotonic increase in risk above a BMI of approximately 20 kg/m^2^ and across the observed range of WC (Figure 2).

**Figure 3:**
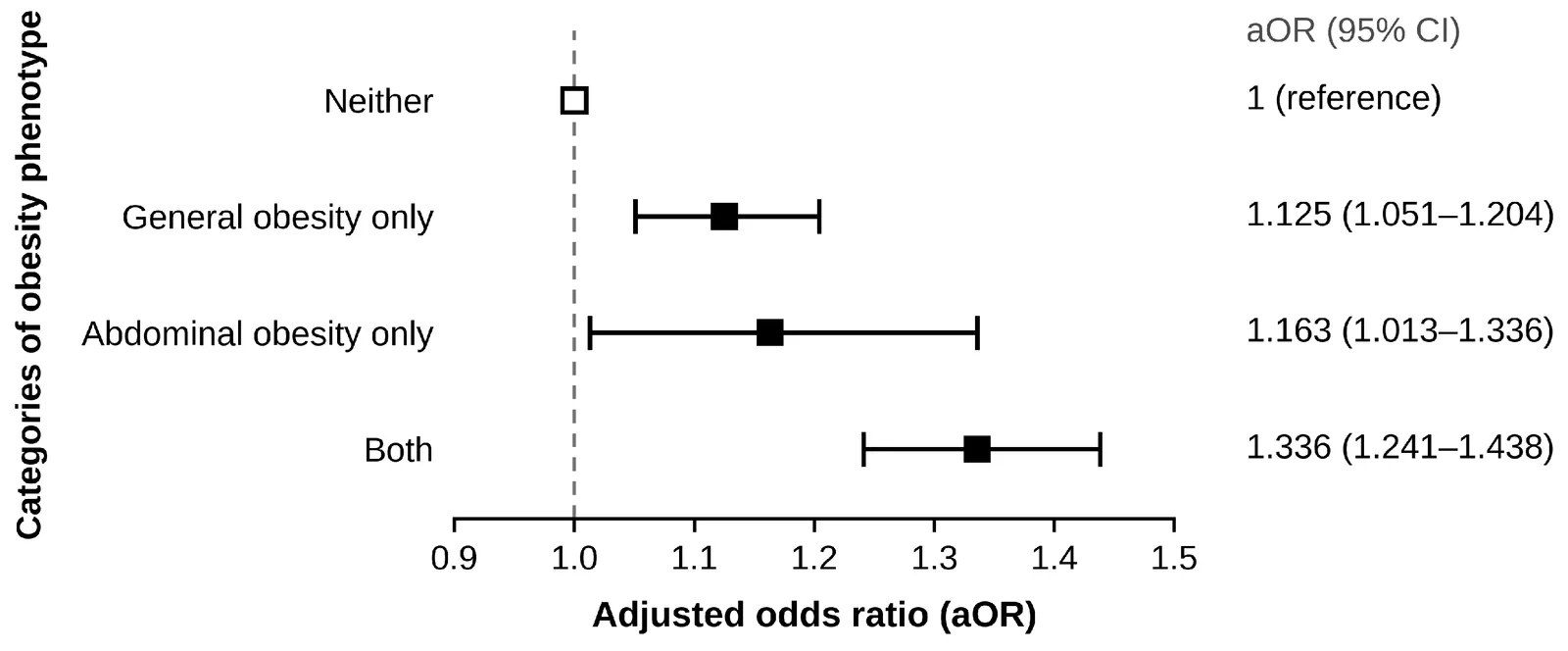
Adjusted odds ratios for oligohydramnios according to joint categories of pre-pregnancy general and abdominal obesity. Squares denote adjusted odds ratios and horizontal lines denote the 95% confidence intervals. The open square denotes the reference group. The dashed vertical line marks an odds ratio of 1.0. Estimates are from Model 2, adjusted for maternal age, smoking status, alcohol consumption, nulliparity, multifetal pregnancy, diabetes mellitus, hypertension, dyslipidemia, chronic kidney disease, SLE/APS, and gynecologic comorbidities (uterine myoma, adenomyosis, endometriosis, PCOS, and history of abortion). Obesity phenotypes were defined according to the Asia-Pacific and Korean Society for the Study of Obesity (KSSO) criteria: general obesity (BMI ≥ 25 kg/m^2^) and abdominal obesity (WC ≥ 85 cm). Abbreviations: BMI, body mass index; WC, waist circumference; SLE, systemic lupus erythematosus; APS, antiphospholipid syndrome; PCOS, polycystic ovarian syndrome.

The joint association of the two phenotypes is shown in Table 3 and Figure 3. The absolute risk of oligohydramnios was highest among women who met criteria for both phenotypes (2.19%), compared with 1.79% among women with neither (Table 3). After adjustment, all three obesity phenotypes were associated with an increased risk relative to women with neither, and the risk was highest for the two combined (1.336, 95% CI 1.241–1.438). The absolute risk difference between women with neither phenotype and those with both was 0.40 percentage points. For women with abdominal obesity alone the unadjusted estimate was below unity and rose above unity after adjustment.

Formal testing provided no evidence of interaction between the two phenotypes. On the additive scale, the relative excess risk due to interaction, the attributable proportion, and the synergy index all included the null value. On the multiplicative scale the product term was not significant (*p* = 0.811). In the unadjusted model the corresponding estimates were of borderline significance (Table 3).

Values in the upper panel are odds ratios with 95% confidence intervals. Absolute risk denotes the proportion of women with oligohydramnios within each phenotype, and risk difference was calculated relative to women with neither phenotype. General obesity was defined as BMI ≥ 25 kg/m^2^ and abdominal obesity as WC ≥ 85 cm. Multiplicative interaction was tested by a product term, entered either with general and abdominal obesity as binary categories or with BMI and WC as continuous variables. Additive interaction was assessed by the relative excess risk due to interaction (RERI), the attributable proportion, and the synergy index. No interaction was evident on either scale in the adjusted models. Model 1 was unadjusted. Model 2, the primary adjusted model, was adjusted for maternal age, smoking status, alcohol consumption, nulliparity, multifetal pregnancy, diabetes mellitus, hypertension, dyslipidemia, CKD, SLE/APS, and gynecologic comorbidities (uterine myoma, adenomyosis, endometriosis, PCOS, and history of abortion). Model 3 comprised Model 2 with additional adjustment for GDM and HDP. Abbreviations: APS, antiphospholipid syndrome; BMI, body mass index; CI, confidence interval; CKD, chronic kidney disease; GDM, gestational diabetes mellitus; HDP, hypertensive disorders of pregnancy; PCOS, polycystic ovarian syndrome; RERI, relative excess risk due to interaction; SLE, systemic lupus erythematosus; WC, waist circumference.

Estimates for general and abdominal obesity were similar in analyses restricted to isolated oligohydramnios and stratified by the examination-to-LMP interval (Table S1), and in the analysis restricted to singleton pregnancies (Table S2).

## 4. Discussion

In this nationwide cohort of 762,104 Korean women, pre-pregnancy general obesity and abdominal obesity were each associated with an increased risk of oligohydramnios, including in the absence of the other phenotype, and the risk was highest among women who met criteria for both. The two measures also identified partly distinct groups of women. Nearly one in four women with abdominal obesity had a BMI below the criterion for general obesity and would have been classified as unexposed had adiposity been assessed by BMI alone, yet their odds of oligohydramnios were increased. Because no interaction was evident on either the additive or the multiplicative scale, assessing both measures extends the group of women identified as being at risk rather than revealing an effect unique to their combination.

Without adjustment, abdominal obesity in the absence of general obesity appeared unassociated with oligohydramnios, and formal tests suggested interaction between the two phenotypes on both scales. After adjustment for pre-pregnancy characteristics, the estimate for abdominal obesity alone reversed direction, with a confidence interval excluding unity, and no interaction remained on either scale.

The successive models further characterize the association. Additional adjustment for GDM and HDP attenuated all estimates, as would be expected if these conditions lie partly along the pathway between pre-pregnancy adiposity and oligohydramnios. Adjustment for fasting glucose and the lipid profile, by contrast, changed the estimates little, so the association is not explained by concomitant dyslipidemia. Adjusted odds ratios increased monotonically across BMI categories. Estimates across WC categories were not strictly monotonic, with the category immediately above the reference close to unity, but spline analysis showed a continuous increase in risk above a BMI of approximately 20 kg/m^2^ and across the observed range of WC. Maternal adiposity relates to oligohydramnios as a graded exposure, and the clinical cut-points used mark positions along a continuum rather than points at which risk begins.

Previous studies of maternal obesity and amniotic fluid volume have yielded inconsistent results. An early-pregnancy cohort reported an increased risk of abnormal amniotic fluid volume among women with obesity [10], whereas other studies found no association with oligohydramnios [11,12,13].

These studies differed in three respects. Most previous studies measured BMI during pregnancy, so that gestational weight gain was incorporated into the exposure. Those were single-center retrospective studies in which a low event rate limited the power to detect associations of the magnitude observed. All defined adiposity by BMI alone. Studies of central adiposity in obstetric populations have relied on ultrasonographic fat measurements with birth weight or cesarean delivery as outcomes [16,17]. The present analysis, to our knowledge, is the first to examine amniotic fluid volume in relation to both general and abdominal obesity, using a measure of central adiposity that is recorded routinely at national health examinations.

Several mechanisms could link maternal adiposity to reduced amniotic fluid volume. Visceral fat is metabolically distinct from subcutaneous fat and is more closely associated with insulin resistance, chronic low-grade inflammation, and oxidative stress [22,23]. These abnormalities have been implicated in impaired trophoblast invasion and placental vascular development [24]. Aquaporins mediate water transport across gestational tissues. Reduced placental vascularization and increased uterine aquaporin-5 expression have been reported in women with obesity [25]. Amniotic fluid volume at term is determined principally by fetal urine production, which depends on fetal renal perfusion [26], and reduced uteroplacental perfusion could therefore reduce amniotic fluid volume. Because general and abdominal obesity were each associated with risk and no interaction was detected, the findings are consistent with two partly overlapping contributions to a shared pathway rather than with an effect that requires both phenotypes to be present. These pathways were not measured in the present study and remain hypotheses to be tested.

HDP were more than twice as frequent among women with oligohydramnios as among women without, a difference large enough to be clinically relevant. GDM and SLE/APS also differed between the groups, by margins that reached statistical significance because of the size of the cohort. HDP are themselves recognized manifestations of impaired placentation.

The associations observed are modest in absolute terms. Women with both phenotypes had an absolute risk of 2.19%, compared with 1.79% among women with neither, a difference of 0.40 percentage points. Therefore, joint classification is better suited to population-level risk stratification than to individual risk prediction. WC is already recorded at national health examinations and requires no additional measurement or cost, so that combining it with BMI is feasible wherever such programs operate. The clinical implications of identifying these women remain uncertain, since the management of oligohydramnios detected at term is itself unsettled [6].

This study has several strengths. The nationwide design provided sufficient events to examine an outcome with a prevalence below 2% while permitting adjustment for a broad range of pre-pregnancy characteristics. BMI and WC were measured by trained staff under a standardized national protocol rather than self-reported. Because these measurements preceded conception, they are not affected by gestational weight gain and reflect maternal adiposity at baseline. Evaluating general obesity and abdominal obesity both separately and in combination provides a framework for risk stratification that a single anthropometric measure does not provide. Interaction between the two phenotypes was evaluated formally on both the additive and the multiplicative scale. Estimates for general and abdominal obesity were consistent across analyses restricted to isolated oligohydramnios and to singleton pregnancies, and across strata defined by the examination-to-conception interval.

Several limitations relating to measurement should be considered. Exposure was assessed at a national health examination performed within two years before the estimated LMP, and anthropometric measures may have changed between examination and conception. Gestational age at delivery was not available. In deliveries extending beyond 280 days, an examination performed shortly before the estimated LMP could fall within the earliest days of gestation, when measurements still closely approximate pre-pregnancy values. Oligohydramnios was ascertained from diagnostic codes without ultrasonographic confirmation of AFI or maximum vertical pocket. Definitions of abnormal amniotic fluid volume vary across national and international guidelines [19], and some diagnostic misclassification is possible. Lifestyle variables were self-reported and are subject to recall bias. None of these errors is expected to differ according to maternal adiposity or the presence of oligohydramnios, so resulting misclassification would be non-differential and more likely to attenuate than to exaggerate the observed associations.

A further set of limitations concerns confounding and the observational design, which permits inference about association but not about causation. Several clinical variables that may influence amniotic fluid volume, including gestational age at diagnosis, membrane rupture, fetal growth restriction, placental insufficiency, maternal hydration status, gestational weight gain, and medication use, are not captured in claims data, and residual confounding cannot be excluded. Although pregnancies with fetal anomalies were excluded, subtle anomalies identifiable only after delivery may not have been fully captured. The cohort was also restricted to women with an eligible health examination, who differed from those without one in parity and in other baseline characteristics. Nevertheless, linkage to the national screening program supplied objectively measured metabolic and anthropometric variables that claims records alone do not contain.

Finally, the findings should be generalized with care. The cohort comprised Korean women who participated in a national screening program, and obesity was defined using cut-points specific to Asian populations, so that the estimates may not transfer directly to other populations or to other diagnostic thresholds. Despite these limitations, the cut-points applied in this study are those used in clinical practice in Korea, so the estimates are directly applicable in the setting from which they derive.

Pre-pregnancy adiposity was associated with oligohydramnios in this nationwide cohort, and the association was evident for abdominal obesity as well as for general obesity. Because a substantial proportion of women with abdominal obesity have a BMI below the obesity threshold, assessment based on BMI alone leaves part of this risk unrecognized. The two measures showed no interaction on either scale, so their joint use widens the group identified as being at risk without implying a distinct combined effect. The higher frequency of HDP among affected women merits further study of the placental contribution to this association. These findings describe an association and do not establish causation, and prospective studies that measure amniotic fluid volume directly are needed before they can inform clinical care.

## Figures and Tables

**Table 1:** Characteristics of the study population according to the presence of oligohydramnios.

Characteristics	Normal Amniotic Fluid (*n* = 748,244)	Oligohydramnios (*n* = 13,860)	*p*-Value
**Interval from examination to LMP, days**			
Mean ± SD	309.77 ± 201.04	306.83 ± 199.93	0.087
Median (Q1–Q3)	283 (140–466)	278 (139–463)	0.107
3 groups			0.333
Within 6 months	240,981 (32.2)	4540 (32.8)	
6 months to 1 year	232,223 (31.0)	4294 (31.0)	
1 to 2 years	275,040 (36.8)	5026 (36.3)	
**Characteristics at delivery**			
Maternal age, years	32.29 ± 3.95	32.17 ± 3.99	<0.001
<25	13,279 (1.8)	274 (2.0)	<0.001
25–29	162,158 (21.7)	3234 (23.3)	
30–34	378,295 (50.6)	6824 (49.2)	
35–39	157,005 (21.0)	2878 (20.8)	
≥40	37,507 (5.0)	650 (4.7)	
Low household income	102,488 (13.7)	1966 (14.2)	0.098
Nulliparity	483,157 (64.6)	10,550 (76.1)	<0.001
Multifetal pregnancy	15,501 (2.1)	180 (1.3)	<0.001
**Pre-pregnancy gynecologic and reproductive history**			
Uterine myoma	47,125 (6.3)	822 (5.9)	0.078
Adenomyosis	9053 (1.2)	202 (1.5)	0.008
Endometriosis	14,420 (1.9)	305 (2.2)	0.021
PCOS	26,499 (3.5)	623 (4.5)	<0.001
History of abortion	181,654 (24.3)	3221 (23.2)	0.005
**Characteristics at the health examination**			
Smoking status			<0.001
Never	684,308 (91.5)	12,498 (90.2)	
Former	32,862 (4.4)	649 (4.7)	
Current	31,074 (4.2)	713 (5.1)	
Alcohol consumption			<0.001
None	402,255 (53.7)	6875 (49.6)	
Mild	310,870 (41.6)	6205 (44.8)	
Heavy	35,119 (4.7)	780 (5.6)	
Regular physical activity	76,863 (10.3)	1599 (11.5)	<0.001
Height, cm	161.22 ± 5.21	161.46 ± 5.29	<0.001
Weight, kg	55.43 ± 8.6	56.13 ± 9.24	<0.001
Pre-pregnancy BMI (kg/m^2^)	21.32 ± 3.1	21.52 ± 3.3	<0.001
General obesity	84,329 (11.3)	1752 (12.6)	<0.001
Waist circumference, cm	70.94 ± 8.08	71.34 ± 8.42	<0.001
Abdominal obesity	48,480 (6.5)	1031 (7.4)	0.001
Systolic BP, mmHg	110.39 ± 11.03	111.1 ± 11.32	<0.001
Diastolic BP, mmHg	69.35 ± 8.27	69.87 ± 8.42	< 0.001
Fasting glucose, mg/dL	88.43 ± 11.9	88.51 ± 11.87	0.399
Total cholesterol, mg/dL	179.85 ± 31.67	180.01 ± 31.45	0.556
HDL-C, mg/dL	63.39 ± 14.08	63.63 ± 14.11	0.044
LDL-C, mg/dL	99.65 ± 27.36	99.6 ± 27.59	0.848
Triglyceride, mg/dL, geometric mean (95% CI)	72.26 (72.18–72.34)	72.07 (71.5–72.64)	0.510
Estimated GFR, mL/min/1.73 m^2^	108.91 ± 18.08	109.95 ± 17.58	<0.001
Diabetes Mellitus	5744 (0.8)	116 (0.8)	0.355
Hypertension	13,247 (1.8)	296 (2.1)	0.001
Dyslipidemia	32,649 (4.4)	595 (4.3)	0.704
Chronic kidney disease	6125 (0.8)	92 (0.7)	0.045
SLE/APS	3950 (0.5)	133 (1.0)	<0.001
**Pregnancy complications**			
GDM	124,130 (16.6)	2515 (18.2)	<0.001
HDP	21,051 (2.8)	908 (6.6)	<0.001

The values are expressed as mean ± standard deviation or number (%). Abbreviations: APS, antiphospholipid syndrome; BMI, body mass index; BP, blood pressure; GDM, gestational diabetes mellitus; GFR, glomerular filtration rate; HDL-C, high-density lipoprotein cholesterol; HDP, hypertensive disorders of pregnancy; LDL-C, low-density lipoprotein cholesterol; PCOS, polycystic ovarian syndrome; SLE, systemic lupus erythematosus.

**Table 2:** Odds ratios for oligohydramnios according to pre-pregnancy body mass index and waist circumference.

					Odds Ratio (95% CI)			
		N	Events	Absolute Risk (%)	Model 1	Model 2	Model 3	Model 4
BMI, kg/m^2^	<18.5	107,703	1873	1.74	0.982 (0.934–1.033)	0.950 (0.903–1.000)	0.959 (0.912–1.009)	0.947 (0.900–0.997)
	18.5–22.9	478,549	8472	1.77	1 (Ref.)	1 (Ref.)	1 (Ref.)	1 (Ref.)
	23.0–24.9	89,771	1763	1.96	1.112 (1.055–1.171)	1.174 (1.114–1.237)	1.150 (1.092–1.212)	1.180 (1.119–1.244)
	25.0–29.9	71,210	1378	1.94	1.095 (1.034–1.160)	1.177 (1.110–1.248)	1.123 (1.059–1.191)	1.188 (1.118–1.262)
	≥30	14,871	374	2.51	1.431 (1.289–1.590)	1.495 (1.343–1.665)	1.337 (1.200–1.491)	1.518 (1.360–1.695)
General obesity	No	676,023	12,108	1.79	1 (Ref.)	1 (Ref.)	1 (Ref.)	1 (Ref.)
	Yes	86,081	1752	2.04	1.139 (1.083–1.198)	1.208 (1.147–1.272)	1.139 (1.081–1.201)	1.206 (1.142–1.273)
WC, cm	<75	557,940	9902	1.77	0.908 (0.852–0.969)	0.831 (0.779–0.887)	0.858 (0.804–0.916)	0.827 (0.775–0.884)
	75.0–79.9	100,610	1873	1.86	0.954 (0.884–1.029)	0.928 (0.860–1.002)	0.941 (0.872–1.016)	0.926 (0.858–1.000)
	80.0–84.9	54,043	1054	1.95	1 (Ref.)	1 (Ref.)	1 (Ref.)	1 (Ref.)
	85.0–89.9	25,606	500	1.95	1.001 (0.899–1.115)	1.022 (0.917–1.138)	1.004 (0.902–1.119)	1.023 (0.919–1.140)
	90.0–94.9	12,958	293	2.26	1.163 (1.021–1.326)	1.214 (1.064–1.385)	1.169 (1.024–1.333)	1.217 (1.067–1.389)
	≥95.0	10,947	238	2.17	1.117 (0.969–1.288)	1.191 (1.032–1.375)	1.113 (0.963–1.285)	1.196 (1.035–1.382)
Abdominal obesity	No	712,593	12,829	1.80	1 (Ref.)	1 (Ref.)	1 (Ref.)	1 (Ref.)
	Yes	49,511	1031	2.08	1.160 (1.088–1.237)	1.281 (1.199–1.369)	1.206 (1.128–1.289)	1.274 (1.190–1.364)

Odds ratios (ORs) and 95% confidence intervals (CIs) for oligohydramnios were estimated according to categories of maternal body mass index (BMI), waist circumference (WC), and the presence of general or abdominal obesity. General obesity was defined as BMI ≥ 25 kg/m^2^; abdominal obesity was defined as WC ≥ 85 cm. Model 1 was unadjusted. Model 2 was adjusted for maternal age, smoking, alcohol consumption, nulliparity, multifetal pregnancy, diabetes mellitus, hypertension, dyslipidemia, chronic kidney disease, SLE/APS, and gynecologic confounders (uterine myoma, adenomyosis, endometriosis, PCOS, history of abortion). Model 3 comprised Model 2 with additional adjustment for GDM and HDP. Model 4 comprised Model 2 with additional adjustment for fasting glucose and the lipid profile. The reference categories were a BMI of 18.5–22.9 kg/m^2^, a WC of 80.0–84.9 cm, and the absence of general or abdominal obesity. Abbreviations: BMI, body mass index; CI, confidence interval; GDM, gestational diabetes mellitus; HDP, hypertensive disorders of pregnancy; ORs, odds ratios; PCOS, polycystic ovarian syndrome; SLE/APS, systemic lupus erythematosus/antiphospholipid syndrome; WC, waist circumference.

**Table 3:** Joint Association of Pre-Pregnancy General and Abdominal Obesity with Oligohydramnios.

General Obesity	Abdominal Obesity					Odds Ratio (95% CI)		
		N	Events	Absolute Risk (%)	Risk Difference (%)	Model 1	Model 2	Model 3
No	No	663,913	11,897	1.79	Ref.	1 (Ref.)	1 (Ref.)	1 (Ref.)
No	Yes	12,110	211	1.74	−0.05	0.972 (0.847–1.115)	1.163 (1.013–1.336)	1.145 (0.997–1.315)
Yes	No	48,680	932	1.91	0.12	1.070 (1.000–1.144)	1.125 (1.051–1.204)	1.077 (1.006–1.153)
Yes	Yes	37,401	820	2.19	0.40	1.229 (1.144–1.320)	1.336 (1.241–1.438)	1.236 (1.147–1.332)
**Interaction between general and abdominal obesity**								
RERI (95% CI)						0.050 (0.0002 to 0.099)	0.012 (−0.039 to 0.062)	0.003 (−0.044 to 0.051)
Attributable proportion (95% CI)						0.043 (0.003 to 0.083)	0.010 (−0.033 to 0.053)	0.003 (−0.040 to 0.046)
Synergy index (95% CI)						5.452 (0.140 to 212.199)	1.077 (0.795 to 1.459)	1.032 (0.675 to 1.577)
*p* for multiplicative interaction								
Categorical specification						0.0497	0.811	0.974
Continuous specification						0.023	0.581	0.888

## Data Availability

The data underlying this study were obtained under license from the National Health Insurance Service (NHIS) of the Republic of Korea and are not publicly available. The data may be accessed from NHIS upon reasonable request, following a formal application process through the NHIS data-sharing portal.

## Supplementary Materials

The following supporting information can be downloaded at: https://www.mdpi.com/article/10.3390/jcm15176652/s1, Table S1: Sensitivity analyses according to the case definition of oligohydramnios and to the interval between health examination and last menstrual period; Table S2: Sensitivity analysis restricted to singleton pregnancies.

## Abbreviations

The following abbreviations are used in this manuscript:

AFI	Amniotic fluid index
APS	Antiphospholipid syndrome
BMI	Body mass index
CI	Confidence interval
GDM	Gestational diabetes mellitus
HDP	Hypertensive disorders of pregnancy
HDL-C	High-density lipoprotein cholesterol
ICD-10	International Classification of Diseases, 10th Revision
IRB	Institutional Review Board
KSSO	Korean Society for the Study of Obesity
LDL-C	Low-density lipoprotein cholesterol
LMP	Last Menstrual Period
NHIS	National Health Insurance Service
NHSE	National Health Screening Examination
OR	Odds ratio
PCOS	Polycystic ovarian syndrome
SLE	Systemic lupus erythematosus
WC	Waist circumference
WHO	World Health Organization
