# Supplementary material for: Pre-Pregnancy Obesity Phenotypes and the Risk of Oligohydramnios: A Large Population-Based Cohort
Source: J Clin Med. 2026 Aug 28;15(17):6652. doi: 10.3390/jcm15176652 (PMC13566875; doi:10.3390/jcm15176652)
Supplement: Supplementary file 1 [file jcm-15-06652-s001.zip › jcm-4443411-supplementary.pdf]

Table S1. Sensitivity analyses according to the case definition of oligohydramnios and to the interval between health examination and last menstrual period.

		N	Events	Absolute risk (%)	Odds ratio (95% CI)		
					Model 1	Model 2	Model 3
**(a) Isolated oligohydramnios (n = 694,535)**							
BMI, kg/m²	< 18.5	97,574	1,485	1.52	0.975 (0.922–1.032)	0.939 (0.887–0.993)	0.946 (0.894–1.002)
	18.5–22.9	435,817	6,797	1.56	1 (Ref.)	1 (Ref.)	1 (Ref.)
	23.0–24.9	82,217	1,395	1.70	1.089 (1.028–1.155)	1.159 (1.093–1.229)	1.138 (1.073–1.207)
	25.0–29.9	65,365	1,145	1.75	1.125 (1.057–1.199)	1.225 (1.149–1.306)	1.176 (1.103–1.255)
	≥ 30.0	13,562	294	2.17	1.400 (1.245–1.576)	1.495 (1.325–1.687)	1.355 (1.199–1.530)
General obesity	No	615,608	9,677	1.57	1 (Ref.)	1 (Ref.)	1 (Ref.)
	Yes	78,927	1,439	1.82	1.163 (1.100–1.230)	1.251 (1.182–1.326)	1.190 (1.123–1.261)
WC, cm	< 75	507,460	7,903	1.56	0.897 (0.836–0.963)	0.810 (0.754–0.870)	0.832 (0.775–0.895)
	75–79.9	92,006	1,509	1.64	0.946 (0.869–1.029)	0.916 (0.842–0.997)	0.927 (0.852–1.009)
	80–84.9	49,510	858	1.73	1 (Ref.)	1 (Ref.)	1 (Ref.)
	85–89.9	23,529	405	1.72	0.993 (0.882–1.119)	1.018 (0.903–1.147)	1.002 (0.889–1.130)
	90–94.9	11,925	243	2.04	1.180 (1.022–1.363)	1.242 (1.075–1.435)	1.201 (1.040–1.388)
	≥ 95	10,105	198	1.96	1.134 (0.970–1.325)	1.227 (1.048–1.436)	1.154 (0.986–1.352)
Abdominal obesity	No	648,976	10,270	1.58	1 (Ref.)	1 (Ref.)	1 (Ref.)
	Yes	45,559	846	1.86	1.177 (1.096–1.263)	1.323 (1.230–1.423)	1.254 (1.165–1.350)
**(b) Health examination within one year before conception (n = 482,038)**							
BMI, kg/m²	< 18.5	66,025	1,141	1.73	0.966 (0.905–1.030)	0.934 (0.875–0.996)	0.942 (0.883–1.005)
	18.5–22.9	302,510	5,410	1.79	1 (Ref.)	1 (Ref.)	1 (Ref.)
	23.0–24.9	57,770	1,141	1.98	1.107 (1.037–1.180)	1.169 (1.096–1.248)	1.148 (1.075–1.225)
	25.0–29.9	46,034	908	1.97	1.105 (1.029–1.186)	1.193 (1.110–1.283)	1.144 (1.063–1.230)
	≥ 30.0	9,699	234	2.41	1.359 (1.190–1.551)	1.443 (1.260–1.652)	1.304 (1.137–1.494)
General obesity	No	426,305	7,692	1.80	1 (Ref.)	1 (Ref.)	1 (Ref.)
	Yes	55,733	1,142	2.05	1.138 (1.069–1.212)	1.216 (1.140–1.297)	1.153 (1.080–1.231)
WC, cm	< 75	351,123	6,262	1.78	0.908 (0.838–0.983)	0.828 (0.764–0.898)	0.853 (0.787–0.925)
	75–79.9	65,360	1,232	1.88	0.960 (0.874–1.055)	0.935 (0.851–1.028)	0.948 (0.863–1.042)
	80–84.9	35,082	688	1.96	1 (Ref.)	1 (Ref.)	1 (Ref.)
	85–89.9	16,099	319	1.98	1.011 (0.884–1.156)	1.024 (0.896–1.172)	1.010 (0.883–1.156)

	90–94.9	7,927	195	2.46	1.262 (1.075–1.482)	1.297 (1.104–1.525)	1.246 (1.060–1.465)
	≥ 95	6,447	138	2.14	1.094 (0.909–1.315)	1.151 (0.955–1.387)	1.076 (0.892–1.297)
Abdominal obesity	No	451,565	8,182	1.81	1 (Ref.)	1 (Ref.)	1 (Ref.)
	Yes	30,473	652	2.14	1.007 (1.004–1.009)	1.013 (1.010–1.015)	1.011 (1.008–1.013)
**(c) Health examination one to two years before conception (n = 280,066)**							
BMI, kg/m²	< 18.5	41,678	732	1.76	1.010 (0.931–1.096)	0.979 (0.902–1.062)	0.989 (0.911–1.073)
	18.5–22.9	176,039	3,062	1.74	1 (Ref.)	1 (Ref.)	1 (Ref.)
	23.0–24.9	32,001	622	1.94	1.120 (1.026–1.222)	1.181 (1.082–1.290)	1.153 (1.056–1.259)
	25.0–29.9	25,176	470	1.87	1.075 (0.974–1.185)	1.146 (1.038–1.267)	1.084 (0.981–1.199)
	≥ 30.0	5,172	140	2.71	1.572 (1.324–1.866)	1.591 (1.333–1.898)	1.397 (1.169–1.671)
General obesity	No	249,718	4,416	1.77	1 (Ref.)	1 (Ref.)	1 (Ref.)
	Yes	30,348	610	2.01	1.139 (1.046–1.241)	1.193 (1.092–1.303)	1.114 (1.018–1.218)
WC, cm	< 75	206,817	3,640	1.76	0.910 (0.817–1.015)	0.837 (0.750–0.934)	0.868 (0.777–0.969)
	75–79.9	35,250	641	1.82	0.941 (0.827–1.071)	0.914 (0.803–1.041)	0.926 (0.813–1.055)
	80–84.9	18,961	366	1.93	1 (Ref.)	1 (Ref.)	1 (Ref.)
	85–89.9	9,507	181	1.90	0.986 (0.824–1.180)	1.015 (0.847–1.215)	0.991 (0.828–1.187)
	90–94.9	5,031	98	1.95	1.009 (0.806–1.264)	1.073 (0.856–1.346)	1.040 (0.829–1.304)
	≥ 95	4,500	100	2.22	1.155 (0.924–1.444)	1.244 (0.991–1.561)	1.161 (0.925–1.457)
Abdominal obesity	No	261,028	4,647	1.78	1 (Ref.)	1 (Ref.)	1 (Ref.)
	Yes	19,038	379	1.99	1.121 (1.008–1.246)	1.248 (1.118–1.393)	1.169 (1.046–1.306)

Isolated oligohydramnios excluded pregnancies with any claim for premature rupture of membranes (O42), fetal growth restriction (O36.5), or post-term pregnancy (O48). Panels (b) and (c) stratify the full cohort by the interval between the health examination and the estimated date of conception.

Model 1 was unadjusted. Model 2, the primary adjusted model, was adjusted for maternal age, smoking status, alcohol consumption, nulliparity, multifetal pregnancy, diabetes mellitus, hypertension, dyslipidemia, chronic kidney disease, SLE/APS, and gynecologic comorbidities (uterine myoma, adenomyosis, endometriosis, PCOS, and history of abortion). Model 3 was additionally adjusted for GDM and HDP.

Abbreviations as in Table 2.

Table S2. Sensitivity analysis restricted to singleton pregnancies.

		N	Events	Absolute risk (%)	Odds ratio (95% CI)		
					**Model 1**	**Model 2**	**Model 3**
General obesity	No	662,305	11,955	1.81	1 (Ref.)	1 (Ref.)	1 (Ref.)
	Yes	84,118	1,725	2.05	1.139 (1.082–1.199)	1.207 (1.146–1.273)	1.139 (1.080–1.201)
Abdominal obesity	No	697,980	12,665	1.81	1 (Ref.)	1 (Ref.)	1 (Ref.)
	Yes	48,443	1,015	2.10	1.158 (1.086–1.235)	1.280 (1.198–1.369)	1.205 (1.127–1.289)

Analysis restricted to singleton pregnancies (n = 746,423; 13,680 events). Multifetal pregnancies (n = 15,681) were excluded.

Model 1 was unadjusted. Model 2, the primary adjusted model, was adjusted for maternal age, smoking status, alcohol consumption, nulliparity, diabetes mellitus, hypertension, dyslipidemia, chronic kidney disease, SLE/APS, and gynecologic comorbidities (uterine myoma, adenomyosis, endometriosis, PCOS, and history of abortion). Model 3 was additionally adjusted for GDM and HDP.

Abbreviations as in Table 2.
